# The first draft genome sequence of *Penicillium adametzioides* FBCC-F1417, isolated from freshwater plant litter in South Korea

**DOI:** 10.1186/s12863-025-01382-7

**Published:** 2025-12-08

**Authors:** Yoeguang Hue, Yebin Nam, Jaeduk Goh, Ki-Tae Kim

**Affiliations:** 1 Department of Plant Medicine, Sunchon National University, Suncheon, Republic of Korea; 2 Prokaryote Research Division, Nakdonggang National Institute of Biological Resources, Sangju, Republic of Korea; 3 Department of Agricultural Life Science, Sunchon National University, Suncheon, Republic of Korea

**Keywords:** Biocontrol agent, Fresh water fungus, Genome, Secondary metabolite, *Penicillium adametzioides*

## Abstract

**Objectives:**

*Penicillium adametzioides* is a phylogenetically distinct and ecologically adaptable member of the genus *Penicillium*. Reports from different isolates and contexts describe postharvest pathogenicity, biocontrol potential, and production of marine-derived metabolites. Because these roles come from different strains and environments, species-level generalizations remain uncertain. To address the lack of genomic data while avoiding overgeneralization, we sequenced and annotated *P. adametzioides* FBCC-F1417, a freshwater plant-litter isolate from Korea, to provide a strain-resolved genomic resource that supports comparative and pangenomic studies across *P. adametzioides* and related taxa.

**Data description:**

We assembled the genome of *P. adametzioides* FBCC-F1417 into 12 contigs totaling 40.47 Mb, with an N50 of 5.16 Mb and GC content of 45.52%. BUSCO analysis showed 99.2% completeness, indicating a high-quality assembly. Gene prediction identified 13,643 protein-coding genes, of which 5,959 proteins (40.7%) were functionally annotated. Repeat analysis showed that 6.62% genome consisted of repetitive elements. Secondary metabolite analysis predicted 86 biosynthetic gene clusters, including NRPS, PKS, terpene, and hybrid types. These features should be interpreted as attributes of FBCC-F1417 rather than definitive species-wide characteristics, and the annotated genome is intended as a reference for downstream comparative and pangenomic analyses that will test which features are conserved across *P. adametzioides*.

## Objective

*Penicillium adametzioides*, first described by S. Abe in 1956, is a phylogenetically distinct species within the genus *Penicillium*, placed in section Sclerotiora [1, 2]. It has been separated from related taxa such as *P. sclerotiorum* and *P. multicolor* through multigene phylogenetic analysis (ITS, cox1, benA, tef1‑α, and cmd) [3]. Originally isolated from soil in Japan, *P. adametzioides* has since been recovered from a wide range of environments, including decayed fruits, insects, and marine ecosystems, highlighting its adaptability and ecological significance [4–6].

Ecologically, it plays multiple roles but critically, these inferences derive from different isolates. While it is known to cause fruit rot in grapes and pomegranates [7, 8], it has also demonstrated potential as a biological control agent. Notably, *P. adametzioides* has been shown to inhibit *Aspergillus carbonarius*, a producer of ochratoxin A, a harmful mycotoxin, making it a promising candidate for pre-harvest treatments in viticulture [9].

From a biotechnological standpoint, this species is also notable for producing a variety of secondary metabolites, including spiroquinazoline alkaloids, bisthiodiketopiperazines, and acorane sesquiterpenes [5, 10]. These compounds have exhibited antimicrobial activity against aqua-pathogenic *Vibrio* species, supporting its potential for applications in aquaculture and natural product discovery.

Although its genome has not yet been publicly released, sequencing *P. adametzioides* would significantly contribute to comparative studies within the genus *Penicillium*, enrich the known pangenome, and offer insights into its biosynthetic capabilities. Its dual role as both a pathogen and biocontrol agent, along with its production of bioactive metabolites, underscores its complex ecological interactions and substantial value in applied research.

## Data description

*P. adametzioides* was isolated from plant litter collected in the Youngsangang River in South Korea (35°8′23.63″N, 126°49′42.63″E) and deposited under accession number FBCC-F1417 in the Freshwater Bioresources Culture Collection (FBCC) at the Nakdonggang National Institute of Biological Resources. For mycological characterization, the isolate was cultured on creatine sucrose agar (CREA), czapek yeast extract agar (CYA), and malt extract agar (MEA) at 25 °C for 7 days, following the protocol by Visagie et al. (2014) [11]. All results below refer specifically to this isolate (FBCC-F1417).

Genomic DNA was extracted from mycelia grown in potato dextrose broth using the DNeasy Plant Mini Kit (Qiagen), yielding 14.952 µg of DNA. DNA quality and quantity were confirmed by PicoGreen dsDNA assay and gel electrophoresis. Whole-genome sequencing was conducted by Macrogen (Seoul, Korea) using the PacBio Sequel and Illumina HiseqX-ten platforms. A total of 14.79 Gb of long-read data (Data File 1 and 2) [12, 13], corresponding to approximately 366 x genome coverage, and 5.00 Gb of short-read data were generated (Data File 3) [14].

*De novo* genome assembly was performed using the HGAP v4.0 with default parameters and Illumina reads were used for error correction via Pilon v1.21 [15, 16]. The final assembly contains 12 contigs totaling 40,469,361 bp, with a GC content of 45.52% (Data File 4) [17, 18]. The longest and shortest contigs were 9,376.3 kb and 98.6 kb, respectively, with an N50 of 5,159.7 kb, and the L50 of 3. This assembly falls within the known size range for *Penicillium* genomes (25.4–46.5 Mbp) [19]. Genome completeness assessed with BUSCO v5.8.2 using the fungi_odb12 dataset (*n* = 1,122) revealed 1,113 complete orthologs, including 1,104 single-copy and 9 duplicates, indicating a completeness of 99.2% (Data File 5) [18, 20].

Gene prediction using MAKER v3.01.03 resulted 13,643 predicted genes (Data File 6), with 14,725 CDS/proteins (Data File 7) [18, 21]. Functional annotation using BLASTP against Swiss-Prot v201806, InterPro v69.0, and eggNOG v4.5.1 revealed 5,959 proteins (40.7%) with known functions based on eggNOG prediction (Data File 8). Repeat analysis via RepeatModeler v2.0.5 and RepeatMasker v4.1.6 identified 2,679,187 bp of repetitive content (6.62% of the genome), composed mainly 5.72% of interspersed repeats, 0.74% of simple repeats, and 0.16% of low complexity repeats (Data File 9) [18, 22, 23].

Secondary metabolite biosynthetic gene clusters (BGCs) were identified with antiSMASH v8.0.2 [24]. In total, 86 BGCs were predicted, comprising 19 type I polyketide synthase clusters, 19 terpene synthetase clusters, 7 non-ribosomal peptide synthases (NRPS), 13 NRPS-like clusters, and 28 hybrid/other metabolite clusters (Data File 10) [18]. Similarity-based annotation detected several high-confidence matches to known bioactive pathways, such as clavaric acid (terpene; farnesyl-protein transferase inhibitor) [25], microperfuranone (NRPS-like; reported from *Aspergillus*) [26], and a choline-related NRPS cluster likely linked to primary metabolism. Additional medium- to low-confidence similarities were found with compounds like burnettiene A, stipitatic acid, and nidulanin A, as well as various other secondary metabolites, suggesting a broad and diverse biosynthetic potential. Overall, these findings highlight *P. adametzioides* as a promising source for natural product discovery and biotechnological applications.

**Table 1:** Overview of data files/data sets

Label	Name of data file/data set	File types (file extension)	Data repository and identifier (DOI or accession number)
Data file 1	PacBio WGS raw data of *P. adametzioides* (FBCC-F1417)	Fastq files (.fastq)	Sequence Read Archive (SRX24773772) [12]
Data file 2	PacBio WGS raw data of *P. adametzioides* (FBCC-F1417)	Fastq files (.fastq)	Sequence Read Archive (SRX24773773) [13]
Data file 3	Illumina WGS raw data of *P. adametzioides* (FBCC-F1417)	Fastq files (.fastq)	Sequence Read Archive (SRX30071030) [14]
Data file 4	*P. adametzioides* draft genome data	Fasta file (.fasta.)	GenBank (JBEBMI000000000.1) [17]
Data file 5	BUSCO results for *P. adametzioides* genome	Portable Document Format file (.pdf)	Figshare (10.6084/m9.figshare.29827163) [18]
Data file 6	*P. adametzioides* gene structural annotation data	Gene Feature Format file (.gff)	Figshare (10.6084/m9.figshare.29827163) [18]
Data file 7	*P. adametzioides* protein sequences data	Fasta file (.fasta)	Figshare (10.6084/m9.figshare.29827163) [18]
Data file 8	*P. adametzioides* protein functional annotation data	MS Excel file (.xlsx)	Figshare (10.6084/m9.figshare.29827163) [18]
Data file 9	*P. adametzioides* repeat analysis results	Zip compressed file (.zip)	Figshare (10.6084/m9.figshare.29827163) [18]
Data file 10	Secondary metabolite biosynthetic gene cluster prediction result for *P. adametzioides*	MS Excel file (.xlsx)	Figshare (10.6084/m9.figshare.29827163) [18]

## Limitations

This study analyzes a single *P. adametzioides* isolate (FBCC-F1417), therefore, all results should be interpreted as strain-specific rather than species-wide. The genome assembly is highly complete by BUSCO (99.2% score), but remains fragmented (12 contigs) and below chromosome-level resolution. Gene prediction was performed without transcriptome evidence, which may affect the accuracy of the predicted gene models. Functional annotations were generated mainly through automated bioinformatics pipelines and publicly available databases (Swiss-Prot, InterPro, eggNOG) without experimental validation of predicted gene functions. Secondary metabolite biosynthetic gene clusters were identified using in silico prediction tools antiSMASH, and their metabolite production has not yet been confirmed through chemical or biological assays. Because accessory genes and especially secondary metabolite BGCs often differ among the same species, and *Penicillium* exhibits a diverse pangenome shaped by gene gain/loss and lateral transfer [19], neither the predicted gene content nor the BGC repertoire reported here should be generalized to all *P. adametzioides*.

## Data Availability

The data described in this Data note can be freely and openly accessed on NCBI Sequence Read Archive under accession numbers SRX24773772, SRX24773773, and SRX30071030, and in GenBank under accession number JBEBMI000000000.1. Genome assembly, annotation, and related analysis files are deposited in Figshare at DOI 10.6084/m9.figshare.29827163. Details are provided in Table 1.

## Abbreviations

ITS: Internal Transcribed Spacer
cox1: Cytochrome c oxidase subunit I
benA: β-tubulin
tef1‑α: Translation elongation factor 1-alpha
cmd: Calmodulin
FBCC: Freshwater Bioresources Culture Collection
CREA: Creatine sucrose agar
CYA: Czapek yeast extract agar
MEA: Malt extract agar
HGAP: Hierarchical Genome Assembly Process
BUSCO: Benchmarking Universal Single-Copy Orthologs
BGCs: Biosynthetic gene clusters
NRPS: Non-ribosomal peptide synthase
